# Epigenetics and animal virus infections

**DOI:** 10.3389/fgene.2015.00048

**Published:** 2015-03-04

**Authors:** Silvia C. Galvan, Alejandro García Carrancá, Jiuzhou Song, Félix Recillas-Targa

**Affiliations:** ^1^Lab Virus and Cancer, Biología Molecular y Biotecnología, Universidad Nacional Autónoma de MéxicoMexico City, Mexico; ^2^Lab Virus and Cancer, Instituto Nacional de Cancerología, Investigación BásicaMexico City, Mexico; ^3^Animal and Avian Sciences, University of MarylandCollege Park, MD, USA; ^4^Genética Molecular, Lab Chromatin Structure and Transcription, Instituto de Fisiología Celular, Universidad Nacional Autónoma de MéxicoMexico City, Mexico

**Keywords:** histones, non-coding RNAs, epigenetics, virus infection, Methylation

Epigenetics, modifications of the genome, heritable during cell division, that do not involve changes in DNA sequences include several mechanisms mainly: histone modifications, DNA methylation and related modifications, non-coding RNAs (ncRNAs) and others that regulate gene expression.

The past two decades has seen an explosion of interest for revealing mechanisms that control epigenetic modifications, mainly based on the influence they have on chromatin structure and their impact in biological processes such as programmed DNA rearrangements, imprinting, germ line silencing, developmentally cued stem cell division, and overall chromosomal stability and identity. It has also become obvious that epigenetics changes are fundamental in the interplay between viruses and their host cells. Generally speaking, when retroviruses and DNA viruses integrate their genomes into the host genome, they can stay latent by silencing their genes or can be productive by activating them, and viral gene expression can be regulated just like as the host. In fact, viral DNA uses host transcription factors as well as epigenetic regulators, in such a way that the effect of viral epigenetic control of its own gene expression also extends to regulate host gene expression. At the same time cells use similar mechanisms, transcription factors and epigenetic modifications, in order to try to eliminate viral infections. In summary, epigenetic mechanisms are involved in most of the virus-cell interactions.

The goal of this special issue is to bring together key experimental and theoretical research linking state-of-the-art knowledge of epigenetic mechanisms involved in regulating virus-cell interactions.

This e-book is a compilation of 12 articles. Two of them are methodological, one on the use of new technologies devoted to identify methylated CpG sites on virus genomes and the other on genome-wide mapping of DNase I hypersenitive sites associated with gene expression. Three articles describe original research involving SV40 minichromosomes, DNA methylation fluctuation and the Toll-like receptor pathways. Seven are review articles, including two mini-reviews on Epigenetic Mechanisms associated to Hepatitis B Virus (HBV) and fundamental topics as DNA methylation, histone modifications and viral strategies against the host immune system in Epstein B Virus; cell differentiation of the immune system as a tool for epigenetic studies; epigenetic mechanisms associated to virotherapy, and finally on the recognition of DNA viruses and cell damage by histones.

The article from Sun et al. (2014), is a methodology article designating the relative advantages of the NGS technology compared to pyrosequencing for studying viral DNA methylation. The analytical procedure they used provided further information related to HPV methylation on a single cell basis, showing that there are HPV 16 genomic sequences in cells which are mostly methylated while in others they are unmethylated (methylation mosaic).

Replication of SV40 minichromosomes can serve as an epigenetic switch in which newly replicated chromatin can be epigenetically modified in response to specific signals such as T-antigen binding to site I. This epigenetic switch seems to ensure that newly replicated minichromosomes do not activate early transcription at late times in infection. In addition, this epigenetic switch may control the relative pool sizes of transcribing, replicating, and encapsidating SV40 minichromosomes. In an original research article, Kallestad et al. (2013), shows that in cells containing SV40 minichromosomes, histone modifications associated with chromatin repression can differ significantly depending upon whether the chromatin is being repressed, undergoes transcription or replication.

The review from Russ et al. (2013), describes advantages of studying the immune system for epigenetic regulation of cell differentiation, in particular how T cell identity or plasticity is controlled. The authors focus some of the key findings and general themes emerging from the studies of T cell differentiation, as well as the utility of the immune system as a tool for studying differentiation and development.

Histones are essential components of chromatin structure, and histone modification plays an important role in various cellular functions including transcription, gene silencing, and immunity. In addition, histones also play distinct roles in extrachromosomal settings. Kobiyama et al. (2013), in their review describe the role of histone H2B as a sensor for dsDNA aberrantly present within the cells. According to the results included, extracellular and extrachromosomal histones alert cells to dangerous situations, such as infection, apoptosis, DNA breaks, and cell injury.

Hepatitis B virus (HBV) infection is a global health problem causing a wide spectrum of liver diseases, including acute and chronic infection. Acute HBV infections either resolve or progress to chronic hepatitis, cirrhosis, and hepatocellular carcinoma. Because for most patients, available therapies do not lead to the termination of HBV infection, improving our understanding of HBV–host interactions is necessary for successful antiviral therapeutic strategies development. The epigenetic mechanisms responsible for viral persistence and clearance during Hepatitis B virus (HBV) replication are addressed in the minireview from Zhang et al. (2013). They outline recent information regarding the epigenetic mechanisms regulating HBV replication and transcription.

The mechanism of latent Epstein Bar Virus (EBV) reactivation *in vivo* is not fully understood, however, it is elicited *in vitro* by treatment of latently infected B cells with chemical and biological reagents. Stimulation of the EBV lytic cascade leads to expression of two viral immediate-early genes, BZLF1 (also known as Zta, EB1, ZEBRA, or Z) and BRLF1 (RtaorR). The BZLF1 protein is a transcriptional activator that resembles the cellular AP1 transcription factors and shares structural similarities to the basic leucine zipper (b-Zip) family of transcription factors. This e-book includes two interesting review articles on BZLF1. The minireview from Sinclair (2013) emphasizes that the epigenetic regulation of EBV differs from the current paradigm, indicating that the presence of CpG methylation in a promoter leads to an absence of expression. The review from Murata and Tsurumi (2013), includes studies on the role of BZLF1 in the switch from EBV latency to the lytic cycle, especially the epigenetic mechanisms involved in BZLF1 gene re-programming during that switch.

A third article related to EBV is the review from Allday (2013) on the control that the human tumor-associated Epstein-Barr virus has on the cell intrinsic defense mechanisms that reduce the risk of neoplasia and cancer (named oncogenic stress responses or OSRs). It describes particularly, how the EBV manipulates the host polycomb group of proteins to control key components of the OSR during normal human B cells transformation into permanent cell lines, by mean of epigenetic repression of transcription.

The article from Forbes et al. (2013) is a review on the interplay between epigenetic modifications that regulate anti-viral responses and how they can be manipulated to improve the therapeutic efficacy of Oncolytic Viruses (OV). The authors add a summary of reports on epigenetic modulation affecting permissibility to virus infections, listing genetic target and their cellular functions, the epigenetic modification, and the cell type involved.

Toll-like receptors (TLRs) constitute an evolutionarily conserved signaling system of the innate immune and inflammatory responses against evolutionarily conserved microbial proteins, lipids, and nucleic acids. The avian genome encodes 10 functional TLRs, located either on the cell surface or within endosomes. The original research article from Kogut et al. (2012), evaluate TLR pathway gene expression differences between heterophils from two lines of chickens, one more resistance than the other to infection with *S. enterica*. The authors profiled the expression of all chicken homologous genes identified in a reference TLR pathway, finding differences mainly between heterophils upon infection with SE.

The original research article from Luo et al. (2012), compare epigenetic features between Marek's disease resistant and susceptible chickens. Main findings indicated some genes have higher promoter methylation in MD-susceptible chickens than resistant ones, and that MDV infection induces expression of DNMT1, DNMT3a, and DNMT3b.

In their original article, He et al. (2014), generated a DNase I hypersensitive sites (DNase I HS) map and gene expression profile for functional analysis, in MDV-transformed CD4+ T-cell line (MSB1). The authors found that DNase I HS sites highly correlate with active genes expression in MSB1 cells.

In summary, in our view main contributions from the articles in this eBook include new findings on already explored topics, as well as new scopes in the field of epigenetic modifications and viral infections.

The new findings include the fact that Next Gen sequencing can be used to analyse HPV 16 DNA methylation mosaic pattern with higher throughput, increased resolution and improved efficiency than pyrosequencing (Sun et al., 2014); an epigenetic switch for histone modifications associated with chromatin repression of SV40 minichromosomes depend upon whether chromatin is undergoing either transcription or replication (Kallestad et al., 2013); Toll-like receptor pathways gene expression differs between resistant and sensitive chicken lines to bacterial infections (Kogut et al., 2012); the fact that MDV infection induces expression of all three methyltransferases genes (DNMT1, DNMT3a, and DNMT3b) in both resistant an sensible chickens, as well as higher methylation of their promoters' in MD-susceptible chickens suggest that epigenetic mechanisms may be involved in modulating resistance to MDV infection in chickens (Luo et al., 2012); DNase I HS sites highly correlate with active genes expression in chicken Marek's disease (He et al., 2014).

New scopes deal with the immune system as model for epigenetic regulation of cell differentiation studies (Russ et al., 2013); the role of histone H2B as a sensor for dsDNA aberrantly present within the cells (Kobiyama et al., 2013); the epigenetic mechanisms involved in HBV persistence and clearance, particularly those regulating viral replication and transcription (Zhang et al., 2013); the epigenetic regulation of EBV, where CpG methylation of viral promoter is required for their expression, which differs from the current paradigm (Sinclair, 2013), and how the tumor-associated EBV manipulates the host polycomb group of proteins to control key components of the oncogenic stress responses (OSR), during the transformation of normal B cells (Allday, 2013); the role of BZLF1 in the switch from EBV latency to the lytic cycle, especially the epigenetic mechanisms involved in BZLF1 gene re-programming (Murata and Tsurumi, 2013); and finally the description of epigenetic modifications regulating anti-viral responses and how they can be used to improve the therapeutic efficacy of oncolytic viruses (Forbes et al., 2013).

## Conflict of interest statement

The authors declare that the research was conducted in the absence of any commercial or financial relationships that could be construed as a potential conflict of interest.
